# Evaluation of the Psychometric Properties of the Functional Assessment of Cancer Therapy-Cervix Questionnaire in Brazil

**DOI:** 10.1371/journal.pone.0077947

**Published:** 2013-10-16

**Authors:** Cristiane Menezes Sirna Fregnani, José Humberto Tavares Guerreiro Fregnani, Maria do Rosário Dias de Oliveira Latorre, Ana Maria de Almeida

**Affiliations:** 1 Graduation Program in Public Health Nursing, College of Nursing, University of São Paulo, Ribeirão Preto, Brazil; 2 Research and Teaching Institute, Barretos Cancer Hospital (IEP-HCB), Barretos, Brazil; 3 Epidemiology Department, School of Public Health, University of São Paulo, São Paulo, Brazil; 4 Department of Maternal-Infant and Public Health Nursing, College of Nursing, University of São Paulo, Ribeirão Preto, Ribeirão Preto, Brazil; University of Missouri Kansas CIty School of Medicine, United States of America

## Abstract

**Background:**

Although cervical cancer is ​​the second most common tumor among Brazilian women, studies that evaluate the quality of life of these women are still scarce. This situation is explained by the lack of specific and validated tools for this purpose in Portuguese (Brazil). The aim of this study was to evaluate the psychometric properties of the Portuguese version of the FACT-CX (Functional Assessment of Cancer Therapy-Cervix) questionnaire in a population of Brazilian women with cervical cancer.

**Methods:**

The psychometric properties of the FACT-CX questionnaire were tested in a sample of 100 women diagnosed with cervical cancer who were previously treated in the Barretos Cancer Hospital. We analyzed the internal consistency (Cronbach's alpha), reproducibility (intraclass correlation coefficient - ICC), confirmatory factor analysis, convergent validity (correlation with the SF-36 questionnaire), and discriminant validity by disease stage and two questions related to self-perception of health was also performed.

**Results:**

The scales had Cronbach´s alpha coefficients ranging from 0.61 to 0.80. However, three scales did not have a statistically significant coefficient greater than 0.70. The ICC ranged from 0.68 to 0.82 and all considered satisfactory. Factor analysis did not generate consistent components. The FACT-G and FACT-CX total scores had good internal consistency and reproducibility, and also correlated well with the General Health and Vitality scales of the SF-36. However, only two FACT-CX scales had a significant correlation with SF-36. Discriminant analysis showed that FACT-CX failed to discriminate groups according to clinical stage but was able to divide the women according to the self-perception of health.

**Conclusion:**

FATC-CX total score had good internal consistency, reproducibility and discriminant validity. In addition, it correlated well with General Health and Vitality scales of SF-36. However, three scales had questionable internal consistency and only two had significant correlation with SF-36.

## Introduction

Cervical cancer is ​​an important public health issue and is the world’s second most common tumor in women. Approximately 530,000 new cases and 270,000 deaths from the disease are recorded worldwide each year [1,2]. In Brazil, cervical cancer is ​​the tumor with the second highest incidence, and approximately 18.000 new cases annually, which corresponds to an incidence rate of 17 cases per 100.000 women [3].

The treatment of cervical cancer has a profound impact on the quality of life of these women and depends on the stage of the disease. Surgical treatment is performed for tumors in the early stages, and this treatment can lead to changes in the quality of life due to the intra- and post-operative morbidity [4-6]. Radiation therapy is the standard treatment for advanced cases and is usually associated with platinum-based chemotherapy [5]. Many studies describe the immediate and late side effects of radiation, which are mainly related to sexual life [7]. The treatment with chemotherapeutic agents induces significant toxicities, some of which are temporary and some of which are permanent [4]. Combination therapy is most often associated with a higher toxicity and more intense side effects [4].

Although cervical cancer is ​​the second most common tumor among Brazilian women [3] , studies that evaluate the quality of life of these women are still rare. The lack of studies in this area is explained by the absence of specific and validated tools for this purpose in Portuguese (Brazil). 

The SF-36 questionnaire (Medical Outcomes Study 36 - Item Short-Form Health Survey) is considered a generic instrument for assessing the quality of life and is easy to administer and understand. The SF-36 questionnaire is a multidimensional questionnaire consisting of 36 items, which are divided into eight scales: physical functioning (PF), role-physical (RP), bodily pain (BP), general health (GH), vitality (VT), social functioning (SF), role-emotional (RE) and mental health (MH) [8]. It is one of the most used questionnaires to evaluate quality of life worldwide, being considered a user-friendly tool and also the gold-standard for all other similar questionnaires [9-12]. Since it was designed as a generic tool, it can be used in clinical practice and in researches as well [13]. However, SF-36 is not able to detect subtle modifications in certain specific setting, such as in cancer patients [14].

The FACIT (Functional Assessment of Chronic Illness Therapy) measurement system is a collection of questionnaires aimed to assess the quality of life of patients with chronic diseases, such as cancer. The first questionnaire developed was the FACT-G, which is used for the general evaluation of the quality of life and is widely used with cancer patients. Specific modules that address issues relevant to specific diseases, treatments, and related conditions were developed to supplement the generic questionnaire. The FACIT questionnaires have been translated into more than 45 languages, which has resulted in more than 50 different questionnaires for specific diseases, conditions and treatments. These questionnaires are easy to apply and interpret with valid and reliable measures [15-17]. In addition to the FACT-G, there is a module dedicated to the uterine cervical cancer (FACT-CX) [18]. Although a Portuguese version of the questionnaire is available, its psychometric properties have never been evaluated in Brazil.

The aim of this study was to evaluate the psychometric properties of the FACT-CX questionnaire in a population of Brazilian women with cervical cancer.

## Patients and Methods

This is a methodological study that was conducted using women who were admitted to the Gynecologic Oncology Department of Barretos Cancer Hospital (Barretos, Brazil) between May and August 2009. One hundred and two women were invited to participate in the study, however two of them refused. Thus, one hundred women were included in the final casuistic. The sample size was estimated according to recommendations performed by Sapnas e Zeller, who suggest 50-100 subjects in order to evaluate the psychometric properties of a questionnaire [19].

 The eligible candidates had a histopathological diagnosis of cervical cancer (*in situ* or invasive) that was previously treated with surgery (conization or radical hysterectomy) and/or chemoradiotherapy. This study was approved by the Research Ethics Committee of the Barretos Cancer Hospital, and all of the women who agreed to participate in the study signed an informed consent form.

Three questionnaires were administered in the form of an interview by a single previously trained interviewer and always in the same order. The first questionnaire involved socio-demographic information (age, marital status, race and education level), and the second was the Short Form 36 (SF-36); the FACT-CX questionnaire was administered last. The clinical data collection was performed according to the information enclosed in the medical records (stage and treatment performed). The understanding level of each question of the FACT-CX questionnaire was verified qualitatively. The women were asked to classify the questions, if necessary, according to one or more of the following: difficult, confusing, and containing difficult or embarrassing words. In order to evaluate the FACT-CX reproducibility, the questionnaire has been sent out to the women´s residence by mail. Ninety three women filled out the form and mailed it back in an average time of 14 days (9 - 19 days). 

 The FACT-CX questionnaire in Portuguese (Brazil) was provided to the authors by the FACIT organization. This questionnaire comprises 27 questions of the FACT-G (generic instrument to assess the quality of life of patients with a chronic disease) and 15 cervical cancer-specific questions, which analyze the symptoms, treatmen, and its sequelae (Additional Concern - CxCS). The FACT-G consists of four scales and contains seven questions about physical well-being (PWB), seven questions about social/family well-being (SWB), six questions about emotional well-being (EWB) and seven questions about functional well-being (FWB). The answers should be based on experiences that occurred in the last seven days. The responses are Likert scaled such that the scores range from 0 to 4 (not at all to very much). A score is assigned to each scale, and, at the end, these scores should be added to obtain a single value. The total score of the questionnaire may vary between 0 and 168. A higher score indicates a better quality of life [16,20,21]. 

The SF-36 questionnaire was previously translated into Portuguese (Brazil) and validated by Ciconelli et al. in 1999 [10]. The final score ranges from 0 to 100 for each scale (a higher score indicates a better quality of life) [8].

 To ensure the quality and consistency of the data, the socio-demographic and clinical information and the questionnaires on the quality of life were entered independently by two typists. The databases were compared, and the discrepancies were corrected *a posteriori*.

The psychometric properties of the FACT-CX instrument were tested through analyses of the reliability (internal consistency and reproducibility), confirmatory factor analysis and the validity (convergent and discriminant). The Cronbach’s alpha coefficient was used to test the internal consistency of the adjusted scales, and values greater than​ or equal to 0.70 were considered suitable [22]. The reproducibility analysis of the FACT-CX was performed by comparing the scores generated by the questionnaire in the first and the second interviews using the intraclass correlation coefficient - ICC (1,1). An ICC equal or greater than 0.40 was considered satisfactory based on Fleiss criteria [23]. 

Also, a factor analysis has been performed in order to confirm whether the questions were able to generate the five known components of FACT-CX (PWB, SWB, EWB, FWB and CxCS). The factor analysis was carried out using a similar strategy proposed by Ding et at. for the Chinese version of FACT-CX validation [18]. The principal component analysis employed an oblique rotation method,forcing a five-factor solution. 

 Convergent validity was assessed by comparing the scores of the FACT-CX with the scores of the SF-36 using Spearman’s correlation coefficient. According to Fayer and Machin [24], a correlation coefficient equal to or above 0.4 is acceptable to evaluate the convergent validity. Three scales of SF-36 (PF, RP and BP) strongly correlate with physical components and another three with mental components (SF, RE and MH). Two of them (GH and VT) have correlations with both components [8]. Based on this principle, we have hypothesized that all FACT-CX scales would correlate with GH and VT scales. In addition, we also supposed that PWB correlate with SF-36 physical components (PR, RP, BP) and EWB with mental components (MH, RE and SF). Since CxCS scale includes physical and emotional issues, a correlation with all SF-36 scales would be expected to occur. Moreover, we hypothesized that SWB could correlate with SF and FWB with PF and SF as well. Confidence intervals (CI95%) for all coefficients (Cronbach, ICC and Spearman) have been calculated. 

 Discriminant validity was analyzed to check the ability of the scales to separate groups with better and poorer qualities of life. Two questions of SF-36 were used as discriminant factors, both related to self-perception of health. These questions were number 1 (“*In general, would you say your health is* …” ) and 2 (“Compared to one year ago, how would you rate your health in general?”). In addition, the clinical stage was also analyzed as a possible discriminant factor (in situ vs. stage I vs. stage II-IV). This method was based on other similar publications [18,25-27]. The comparison of the scores was performed using the Mann-Whitney or the Kruskal-Wallis tests depending on the number of categories compared. 

Scores were calculated according to FACIT and SF-36 manuals. SPSS (version 20) or MedCalc (version 11) were used in all statistical analyzes. The level of significance was set at 5%.

## Results

Table 1 provides a description of the sample. The population of this study consisted of women with a mean age of 42.4 years (SD = 11.8), mostly white, married, and with a low level of education.

**Table 1:** Characteristics of the women sampled (n = 100).

Variable	Category	Number
Race	White	72
	Black	28
Marital status	Married	70
	Divorced	17
	Single	10
	Widowed	3
Education	Up to 8 years	70
	> 8 years	30
Clinical stage	in situ	45
	IA	06
	IB	17
	II	17
	III	13
	IV	02
Type of treatment	Surgery	56
	Surgery, chemotherapy, and radiotherapy	44
TOTAL		100

The assessment of the understanding of each question (Table 2) revealed that none of the questions was considered to contain difficult or embarrassing words, but most of the complaints were due to the question being either confusing or difficult to understand. The questions related to the PWB scale presented the highest number of complaints.

**Table 2:** Number of respondents who reported some complaint concerning the FACT-CX questionnaire, as determined in their evaluation of the level of understanding of each question.

		Number of complaints for each question			
FACT-CX Scales	Items	Difficult to Understand	Confusing	Difficult Words	Embarrassing
PWB	GP1 – I have a lack of energy.	-	6	-	-
	GP3 – Because of my physical condition, I have trouble meeting the needs of my family.	-	2	-	-
	GP5 – I am bothered by the side effects of treatment.	2	3	-	-
	GP7 – I am forced to spend time in bed.	-	2	-	-
SWB	GS1 – I feel close to my friends.	-	2	-	-
	GS4 – My family has accepted my illness.	-	3	-	-
	GS5 – I am satisfied with family communication about my illness.	-	2	-	-
EWB	GE3 – I am losing hope in the fight against my illness.	-	3	-	-
FWB	GF2 – My work (include work at home) is fulfilling.	-	2	-	-
CxCS	CX1 – I am bothered by discharge or bleeding from my vagina.	-	3	-	-
	B4 – I feel sexually attractive.	1	1	-	-
	CX5 – I am afraid that the treatment may harm my body.	-	2	-	-

PWB: Physical well-being, SWB: Social/Family well-being, EWB: Emotional well-being, FWB: Functional well-being, CxCS: Additional Concerns.

Table 3 shows the statistics of the scores obtained for each scale and the corresponding Cronbach’s alpha coefficients. Three scales did not have a statistically significant coefficient greater than 0.70: SWB = 0.75 (95%CI: 0.67-0.82), EWB = 0.61 (95%CI: 0.47-0.72) and CxCS = 0.75 (CI95%: 0.67-0.81). Alpha coefficients for FACT-CX and FACT-G total score were respectively 0.90 (95%CI: 0.87-0.92) and 0.88 (95%CI: 0.85-0.92). The ICC which evaluated the reproducibility of the scales of the FACT-CX questionnaire ranged from 0.68 to 0.82 and all considered satisfactory. A correlation matrix among all individual items of the questionnaire is shown in the appendix (Table S1). 

**Table 3:** Descriptive analysis of the scales of the FACT-CX questionnaire, the corresponding Cronbach’s alpha coefficients, and the intraclass correlation coefficients.

	Mean (SD)	Median	Minimum - Maximum	Cronbach’s alpha	ICC (95%CI)
PWB	23.4 (4.6)	25.0	10 - 28	0.78 (0.70-0.84)	0.71 (0.59-0.79)
SWB	19.0 (5.8)	19.0	1 - 28	0.75 (0.67-0.82)	0.74 (0.64-0.82)
EWB	18.2 (4.2)	18.5	5 - 24	0.61 (0.47-0.72)	0.68 (0.56-0.78)
FWB	20.4 (5.2)	20.0	8 - 28	0.80 (0.73-0.85)	0.71 (0.59-0.80)
CxCS	43.9 (8.7)	45.0	15 - 60	0.75 (0.67-0.81)	0.82 (0.73-0.88)
FACT-G	81.1 (15.7)	83.0	32 - 108	0.88 (0.85-0.92)	0.81 (0.73-0.87)
FACT-CX	110.4 (25.6)	114.1	34.1 - 148.2	0.90 (0.87-0.92)	0.84 (0.76-0.89)

95%CI: 95% Confidence interval. PWB: Physical well-being, SWB: Social/Family well-being, EWB: Emotional well-being, FWB: Functional well-being, CxCS: Additional Concerns, ICC: Intraclass correlation coefficient, FACT-G: PWB + SWB + EWB + FWB, FACT-CX: FACT-G + CxCS.

Factor analysis can be seen in table 4. The solution accounted for 46.2% of the explained variance. PWB and SWB were mostly compared to the first and second components, having them the highest percentage of variance. However, the other scales did not generate consistent components. 

**Table 4:** Factor Analysis of FACT-CX questionnaire.

		1	2	3	4	5
Physical well-being (PWB)						
GP1	I have a lack of energy	0.523	-0.063	-0.003	0.111	-0.279
GP2	I have nausea	0.333	0.100	0.241	0.139	-0.022
GP3	Because of my physical condition0. I have trouble meeting the needs of my family	0.597	0.139	0.200	0.031	-0.026
GP4	I have pain	0.540	-0.034	0.202	0.142	-0.354
GP5	I am bothered by side effects of treatment	0.168	-0.058	0.571	0.116	0.066
GP6	I feel ill	0.700	-0.028	-0.043	0.173	-0.032
GP7	I am forced to spend time in bed	0.579	-0.029	-0.057	0.108	-0.145
Social/family well-being (SWB)						
GS1	I feel close to my friends	0.076	-0.642	0.001	-0.091	0.114
GS2	I get emotional support from my family	-0.112	-0.741	0.076	-0.148	-0.146
GS3	I get support from my friends	-0.088	-0.714	0.136	-0.152	-0.095
GS4	My family has accepted my illness	-0.098	-0.751	-0.066	0.171	0.092
GS5	I am satisfied with family communication about my illness	-0.057	-0.766	-0.039	0.122	-0.014
GS6	I feel close to my partner (or the person who is my main support)	0.085	-0.400	0.094	-0.250	-0.248
GS7	I am satisfied with my sex life	0.331	0.051	0.487	-0.257	-0.157
Emotional well-being (EWB)						
GE1	I feel sad	0.372	-0.003	0.287	0.136	-0.395
GE2	I am satisfied with how I am coping with my illness	0.193	-0.428	0.128	0.083	0.374
GE3	I am losing hope in the fight against my illness	0.301	0.031	-0.007	-0.023	0.090
GE4	I feel nervous	0.073	-0.069	0.387	-0.017	-0.507
GE5	I worry about dying	0.072	-0.097	0.098	0.550	-0.030
GE6	I worry that my condition will get worse	0.373	-0.069	0.035	0.587	-0.045
Functional well-being (FWB)						
GF1	I am able to work (include work at home)	0.733	-0.160	0.093	-0.126	0.069
GF2	My work (include work at home) is fulfilling	0.603	-0.257	-0.002	0.037	0.044
GF3	I am able to enjoy life	0.425	-0.367	0.057	-0.076	0.236
GF4	I have accepted my illness	0.076	-0.640	0.051	0.277	0.422
GF5	I am sleeping well	0.145	-0.382	-0.072	0.134	-0.451
GF6	I am enjoying the things I usually do for fun	0.307	-0.552	-0.146	-0.025	-0.040
GF7	I am content with the quality of my life right now	0.427	-0.427	-0.095	0.162	-0.266
Additional Concerns (CxCS)						
Cx1	I am bothered by discharge or bleeding from my vagina	-0.028	-0.030	0.188	0.548	-0.306
Cx2	I am bothered by odor coming from my vagina	-0.146	0.010	0.453	0.417	-0.247
Cx3	I am afraid to have sex	-0.120	-0.143	0.527	-0.100	-0.148
B4	I feel sexually attractive	0.390	-0.062	0.305	-0.223	0.217
Cx4	My vagina feels too narrow or short	-0.019	-0.160	0.528	0.281	-0.049
BMT7	I have concerns about my ability to have children	0.023	0.006	-0.038	0.649	0.003
Cx5	I am afraid the treatment may harm my body	0.129	0.049	0.202	0.566	0.308
BL4	I am interested in sex	0.284	-0.173	0.253	-0.420	0.031
C7	I like the appearance of my body	0.158	-0.191	0.327	-0.119	0.017
Cx6	I am bothered by constipation	0.139	0.167	0.120	0.218	-0.442
C6	I have a good appetite	0.600	0.012	-0.177	-0.037	-0.099
BL1	I have trouble controlling my urine	-0.140	0.044	0.697	-0.024	-0.116
BL3	It burns when I urinate	-0.031	0.089	0.830	0.147	0.159
Cx7	I have discomfort when I urinate	0.098	0.156	0.724	0.211	0.235
HN1	I am able to eat the foods that I like	0.439	-0.040	0.089	-0.007	0.292
Rotation Sums of Squared Loadings (Total)		8.862	4.209	2.239	2.179	1.934
Variance Contribution = 46.2%		21.1%	10.0%	5.3%	5.2%	4.6%

The convergent validity analysis is demonstrated in Tables 5 and 6. PWB and EWB scales had the best correlation with the correspondent SF-36 scales. No correlation was found between SWB, FWB and CxCS scales and SF-36. Regarding FACT-G and FACT-CX total scores, both were significantly correlated with GH and VT. In addition, BD also correlated with CxCS. 

**Table 5:** Correlation coefficients between FACT-CX and SF-36 (convergent validity).

	FACT-CX Scales						
SF-36 Scales	PWB	SWB	EWB	FWB	CxCS	FACT-G	FACT-Cx
	r_s_ (95%CI)	r_s_ (95%CI)	r_s_ (95%CI)	r_s_ (95%CI)	r_s_ (95%CI)	r_s_ (95%CI)	r_s_ (95%CI)
Physical functioning (PF)	0.69 (0.57-0.78)	0.21 (0.02-0.39)	0.40 (0.22-0.55)	0.32 (0.13-0.49)	0.36 (0.18-0.52)	0.48 (0.31-0.62)	0.49 (0.33-0.63)
Role-physical (RP)	0.50 (0.34-0.63)	0.18 (-0.02-0.36)	0.21 (0.02-0.39)	0.29 (0.10-0.46)	0.16 (-0.04-0.35)	0.36 (0.18-0.52)	0.34 (0.15-0.50)
Bodily pain (BP)	0.67 (0.55-0.77)	0.22 (0.03-0.40)	0.55 (0.40-0.67)	0.32 (0.13-0.49)	0.45 (0.28-0.59)	0.54 (0.39-0.67)	0.58 (0.43-0.70)
General health (GH)	0.71 (0.60-0.80)	0.33 (0.14-0.49)	0.63 (0.50-0.74)	0.47 (0.30-0.61)	0.40 (0.22-0.55)	0.66 (0.53-0.76)	0.64 (0.51-0.74)
Vitality (VT)	0.68 (0.56-0.77)	0.21 (0.02-0.40)	0.57 (0.42-0.69)	0.39 (0.21-0.54)	0.44 (0.27-0.59)	0.55 (0.40-0.67)	0.59 (0.45-0.70)
Social functioning (SF)	0.51 (0.35-0.64)	0.15 (-0.05-0.34)	0.41 (0.23-0.56)	0.29 (0.10-0.46)	0.27 (0.08-0.44)	0.42 (0.24-0.57)	0.44 (0.27-0.59)
Role-emotional (RE)	0.47 (0.30-0.61)	0.13 (-0.07-0.32)	0.20 (0.00-0.38)	0.29 (0.10-0.46)	0.14 (-0.06-0.33)	0.34 (0.15-0.50)	0.32 (0.13-0.49)
Mental health (MH)	0.53 (0.37-0.66)	0.24 (0.05-0.42)	0.55 (0.40-0.67)	0.32 (0.13-0.49)	0.34 (0.15-0.50)	0.48 (0.31-0.62)	0.49 (0.33-0.63)

r_s_ = Spearman’s correlation coefficient 95%CI: 95% Confidence interval. PWB: Physical well-being, SWB: Social/Family well-being, EWB: Emotional well-being, FWB: Functional well-being, CxCS: Additional Concerns, FACT-G: PWB + SWB + EWB + FWB, FACT-CX: FACT-G + CxCS.

**Table 6:** Expected correlations between FACT-CX and SF-36 scales (convergent validity).

FACT-CX	SF-36	Number of expected correlations between FACT-CX and SF-36 scales	Number of significant correlations (*) found between FACT-CX and SF-36 scales	
PWB	PR, RP, BP, GH, VT	5	4	80.0%
SWB	SF, GH, VT	3	0	0.0%
EWB	MH, RE, SF, GH, VT	5	3	60.0%
FWB	PF, SF, GH, VT	4	0	0.0%
CxCS	PR, RP, BP, GH, VT, MH, RE, SF	8	0	0.0%
FACT-G	PR, RP, BP, GH, VT, MH, RE, SF	8	2	25.0%
FACT-Cx	PR, RP, BP, GH, VT, MH, RE, SF	8	3	37.5%

(*)Significant correlation means a Spearman´s coefficient greater than 0.40, with the lower limit of its confidence interval equal or greater than 0.40.

PWB: Physical well-being, SWB: Social/Family well-being, EWB: Emotional well-being, FWB: Functional well-being, CxCS: Additional Concerns, FACT-G: PWB + SWB + EWB + FWB, FACT-CX: FACT-G + CxCS, PF: Physical functioning, RP: Role-physical, BP: Bodily pain, GH: General health, VT: Vitality, SF: Social functioning, SF: Social functioning, RE: Role-emotional, MH: Mental health.

Table 7 illustrates the discriminant validity analysis. FACT-CX was able to discriminate groups according to two questions regarding the self-perception of health (questions 1 and 2 of SF-36), but did not in relation to clinical stage (in situ vs. stage I vs. stage II-IV). 

**Table 7:** Comparison of means of FACT-CX scales according to the clinical stage and SF-36 questions number 1 and 2 (discriminant validity).

	Question #1 , SF36: “In general, would you say your health is …”				Question #2, SF36: “Compared to one year ago, how would you rate your health in general now?”			Clinical stage			
	Excellent/ Very good	Good	Fair/Poor	P value	Better	Equal or worse	P value	In situ	Stage I	Stage II - IV	P value
	(n=55)	(n=39)	(n=6)		(n=55)	(n=45)		(n=45)	(n=23)	(n=32)	
	Mean (SD)	Mean (SD)	Mean (SD)		Mean (SD)	Mean (SD)		Mean (SD)	Mean (SD)	Mean (SD)	
PWE	25.1 (3.3)	22.2 (4.7)	16.0 (4.4)	< 0.001	24.7 (3.7)	21.9 (5.0)	0.001	23.7 (4.5)	23.5 (5.0)	23.1 (4.5)	0.571
SWB	19.6 (5.9)	18.7 (5.7)	15.7 (5.2)	0.262	20.1 (5.9)	17.6 (5.5)	0.051	19.7 (6.1)	19.1 (6.7)	18.0 (4.6)	0.732
EWB	19.6 (3.6)	17.0 (4.1)	13.5 (5.0)	0.001	19.5 (3.8)	16.7 (4.2)	0.001	17.5 (4.2)	18.5 (4.8)	19.1 (3.6)	0.952
FWB	21.2 (4.8)	20.4 (4.9)	11.8 (2.6)	0.001	21.5 (5.0)	19.0 (5.2)	0.022	20.2 (5.7)	20.5 (5.6)	20.5 (4.4)	0.824
CxCS	45.1 (8.8)	43.3 (7.4)	36.5 (12.8)	0.158	45.2 (9.2)	42.4 (8.0)	0.029	44.0 (8.1)	45.4 (9.9)	42.8 (8.8)	0.114
FACT-G	85.6 (13.7)	78.4 (15.5)	57.0 (6.2)	< 0.001	85.9 (14.8)	75.2 (14.9)	< 0.001	81.1 (17.1)	81.6 (18.6)	80.6 (11.1)	0.550
FACT-CX	130.7 (20.0)	121.7 (20.6)	93.5 (15.8)	< 0.001	131.1 (21.1)	117.5 (20.5)	< 0.001	125.1 (22.6)	127.0 (27.0)	123.4 (16.5)	0.294

PWB: Physical well-being, SWB: Social/Family well-being, EWB: Emotional well-being, FWB: Functional well-being, CxCS: Additional Concerns, FACT-G: PWB + SWB + EWB + FWB, FACT-CX: FACT-G + CxCS.

## Discussion

To the best of our knowledge, this is the first published validation of the Portuguese version of the FACT-CX questionnaire. In general, the questionnaire showed a good level of understanding and good reproducibility. The lack of studies evaluating the psychometric properties of the FACT-CX questionnaire made it difficult to perform some comparisons. The only study that we found in the literature on this topic was that conducted by Ding et al., who validated the questionnaire in 400 Chinese women with cervical cancer [18]. According to the authors, the Chinese version of the questionnaire had acceptable internal consistency and obtained a good discrimination of groups. In 2010, Fernandes and Kimura used the non-validated version of the Portuguese (Brazil) FACT-CX questionnaire to assess the quality of life in 149 women with cervical cancer. The only psychometric property assessed by the authors was the internal consistency using Cronbach's alpha coefficient [28]. The mean scores of the FACT-CX scales observed in the present study were similar to those found by Fernandes and Kimura.

A major limitation of the present study is that the sample consisted mostly of women with a low educational level. Only 30% of the participants had more than 8 years of education. Part of this limitation can be observed in the understanding level of the questions. Although none of the women complained that the questions contained difficult words, the complaints were related to the lack of full understanding of the question (difficult to understand or confusing question). This lack of understanding was particularly highlighted by the GP1 question (“I have a lack of energy”) and the GP5 question (“I am bothered by side effects of treatment”), which presented the highest number of complaints. These understanding difficulties may be related to the fact that the Barretos Cancer Hospital serves women from various regions of the country, where certain expressions and phrases may not be of common use in daily life. It is also interesting to note that none of the participants reported any difficulty in answering the questions because of embarrassment caused by certain words. Ding et al. observed that the patients found it difficult to answer the questions that addressed sexuality, and this difficulty may be related to the conservative culture of that country [18]. 

 The low level of education may have introduced some bias into the validation of the questionnaire. However, the sample of participants of the present study perfectly reflects the reality of most Brazilian public hospitals, especially the women with cervical cancer. Thus, the validation of the FACT-CX in this context could be applied with relative ease to the Brazilian situation.

Three scales (SWB, EWB and CxCS) did not have Cronbach’s alpha values significantly higher than 0.70, which suggests that these scale items are not measuring the same feature [22]. But we should consider that the sample size may not have been enough to evaluate two scales: SWB e CxCS had alpha values greater than 0.70, but the lower limit of the confidence interval was below 0.70. Probably, a larger sample size could narrow the confidence interval, making the alpha values significantly higher than 0.70. In the study conducted by Fernandes and Kimura and in the study conducted by Ding et al., the internal consistency values ​​found for the CxCS scale were also below 0.70 [18,28]. The analysis of the items in the scale of CxCS showed that some of the items would not have been included *a priori* into the same scale [29]. Some of the questions are clearly related to sexuality (CX3, B4, BL4), whereas others are related to body image (C7), symptoms of the disease (CX1, CX2), and adverse effects after treatment (CX4, BMT7, CX5, CX6, BL1, BL3, CX7, HN1, C6). These results suggest that CxCS scale should be assessed in subscales or included in other scales, and not as a general one. 

 The EWB scale of the FACT-G, which comprises 6 items, showed the lowest internal consistency. This result is in disagreement with the coefficients reported by Ding and colleagues [18] and Fernandes and Kimura [28], who found values ​​of 0.76 and 0.78, respectively. However, other publications also cited problems in the internal consistency of this scale with ​Cronbach’s alpha values in the range of 0.33 to 0.60 [30-32]. The low internal consistency observed in the EWB scale may be explained by the difficulty of understanding one or more of the items. Specifically in this scale, three women said that they found the GE3 question (“I am losing hope in the fight against my illness”) confusing. However, the data suggest that there may be other items on this scale that were not correctly understood by the women. Another explanation for this low consistency, which was proposed by Garland et al. [32], is the fact that the GE3 question is encoded in reverse compared with the other questions in the scale. These authors also suggest that patients who had already been treated would be free from the morbid influence of the anxiety associated with the initial diagnosis and the treatment proposal, which would obviously make the assessment of the EWB scale difficult. In fact, all of the women who participated in the present study had undergone treatment of the tumor, which supports the assumption proposed by Garland et al. 

The FACT-CX questionnaire showed good reproducibility through the evaluation of the intraclass correlation coefficients. Other studies that tested the reproducibility of the FACT-G questionnaire and their specific modules confirm the results of the present study [25,33-37].

The factor analysis showed, in contrast to earlier findings [18], inconsistent results. It was expected that FACT-CX would be able to generate five consistent components. However, only PWB and SWB were clearly related to two separate components. The other scales did not generate any clear factor. These results might be explained by the sample size. Nunnally recommended having 10 times as many variables in the questionnaire to run a factor analysis [38]. Thus, considering that FACT-CX has 42 variables, it would be necessary to have more than 400 women in order to run a consistent analysis. Indeed, Ding et al. [18] were able to confirm the four components of FACT-G (27 questions) with a sample size of 400 Chinese women. 

 The FACT-G and FACT-CX total scores correlated well with the GH and VT scales of the SF-36, as expected to be. However, some caveats should be made. SWB, FWB and CxCS did not have any correlation with the SF-36 scales. The review of the questions of both questionnaires revealed that the FACT-CX questionnaire investigates the social items more thoroughly through seven questions, whereas the SF-36 does so superficially through only two questions. A possible explanation for lack of correlation between CxCS and SF-36 scales might be that CxCS addresses issues of sexuality, which are not covered by the other questionnaire. Moreover, SF-36 is a generic questionnaire which theoretically is unable to detect some specific features about cervical cancer. Since the proposal of the FACT-CX and SF-36 are different, some correlations might not be feasible. 

 The analysis of the discriminant validity of the FACT-CX questionnaire showed that this questionnaire failed to discriminate the groups according to clinical stage but was able to divide the women according to the self-perception of health. Like us, Ding et al. also found that the questionnaire did not exhibit discriminating capability according to the clinical stage [18]. It is not surprising that the questionnaire was not able to discriminate women according to clinical stage. All women did not have any oncologic disease or specific symptoms at the moment of the study. 

In summary, this is the first study that evaluated the psychometric properties of the FACT-CX questionnaire in Portuguese (Brazil). FACT-CX total score had good internal consistency, reproducibility and discriminant validity. In addition, it correlated well with GH and VT scales of SF-36. Regarding the five scales, all had satisfactory reproducibility, but three scales (PWB, SWB and CxCS) had questionable internal consistency. In general, the scales presented good discriminant validity, but only PWB and EWB had significant correlation with SF-36. Finally, factor analysis did not confirm the five components of FACT-CX. However, further studies are recommended to conclude that FACT questionnaire is valid and reliable. In addition, FACT-CX should be evaluated in different populations from the cultural and socioeconomic point of view and with a larger sample to run a consistent factor analysis. 

## Supporting Information

Table S1 **Correlation matrix among all individual items of FACT-CX.**
(PDF)
