# Supplementary material for: Evaluation of the Psychometric Properties of the Functional Assessment of Cancer Therapy-Cervix Questionnaire in Brazil
Source: PLoS One. 2013 Oct 16;8(10):e77947. doi: 10.1371/journal.pone.0077947 (PMC3797824; doi:10.1371/journal.pone.0077947)
Supplement: Table S1 — Correlation matrix among all individual items of FACT-CX. (PDF) [file pone.0077947.s001.pdf]

Correlations

			GP1	GP2	GP3	GP4	GP5	GP6	GP7	GS1	GS2	GS3	GS4	GS5	GS6	GS7
Spearman's rho	GP1	Correlation Coefficient	1,000	,301**	,480**	,454**	,223*	,356**	,308**	,127	,189	,185	,128	,126	,145	,154
		Sig. (2-tailed)	.	,002	,000	,000	,026	,000	,002	,209	,059	,066	,203	,212	,150	,126
		N	100	100	100	100	100	100	100	100	100	100	100	100	100	100
	GP2	Correlation Coefficient	,301**	1,000	,272**	,383**	,249*	,317**	,302**	,114	-,063	,034	,176	,150	-,013	,153
		Sig. (2-tailed)	,002	.	,006	,000	,013	,001	,002	,257	,532	,740	,080	,136	,896	,128
		N	100	100	100	100	100	100	100	100	100	100	100	100	100	100
	GP3	Correlation Coefficient	,480**	,272**	1,000	,379**	,308**	,369**	,339**	,145	,049	,063	,104	,148	,216*	,309**
		Sig. (2-tailed)	,000	,006	.	,000	,002	,000	,001	,150	,631	,534	,302	,142	,031	,002
		N	100	100	100	100	100	100	100	100	100	100	100	100	100	100
	GP4	Correlation Coefficient	,454**	,383**	,379**	1,000	,346**	,437**	,399**	,190	,203*	,229*	,012	,182	,112	,280**
		Sig. (2-tailed)	,000	,000	,000	.	,000	,000	,000	,059	,043	,022	,905	,069	,268	,005
		N	100	100	100	100	100	100	100	100	100	100	100	100	100	100
	GP5	Correlation Coefficient	,223*	,249*	,308**	,346**	1,000	,277**	,296**	,116	,063	,185	,232*	,188	,253*	,327**
		Sig. (2-tailed)	,026	,013	,002	,000	.	,005	,003	,249	,530	,065	,020	,061	,011	,001
		N	100	100	100	100	100	100	100	100	100	100	100	100	100	100
	GP6	Correlation Coefficient	,356**	,317**	,369**	,437**	,277**	1,000	,484**	,204*	,121	,133	,102	,179	,181	,278**
		Sig. (2-tailed)	,000	,001	,000	,000	,005	.	,000	,041	,232	,187	,313	,074	,072	,005
		N	100	100	100	100	100	100	100	100	100	100	100	100	100	100
	GP7	Correlation Coefficient	,308**	,302**	,339**	,399**	,296**	,484**	1,000	,219*	,221*	,157	,235*	,243*	,257**	,248*
		Sig. (2-tailed)	,002	,002	,001	,000	,003	,000	.	,028	,027	,120	,019	,015	,010	,013
		N	100	100	100	100	100	100	100	100	100	100	100	100	100	100
	GS1	Correlation Coefficient	,127	,114	,145	,190	,116	,204*	,219*	1,000	,394**	,679**	,375**	,366**	,218*	,081
		Sig. (2-tailed)	,209	,257	,150	,059	,249	,041	,028	.	,000	,000	,000	,000	,029	,424
		N	100	100	100	100	100	100	100	100	100	100	100	100	100	100
	GS2	Correlation Coefficient	,189	-,063	,049	,203*	,063	,121	,221*	,394**	1,000	,603**	,455**	,623**	,480**	,086
		Sig. (2-tailed)	,059	,532	,631	,043	,530	,232	,027	,000	.	,000	,000	,000	,000	,397
		N	100	100	100	100	100	100	100	100	100	100	100	100	100	100
	GS3	Correlation Coefficient	,185	,034	,063	,229*	,185	,133	,157	,679**	,603**	1,000	,394**	,490**	,368**	,083
		Sig. (2-tailed)	,066	,740	,534	,022	,065	,187	,120	,000	,000	.	,000	,000	,000	,413
		N	100	100	100	100	100	100	100	100	100	100	100	100	100	100
	GS4	Correlation Coefficient	,128	,176	,104	,012	,232*	,102	,235*	,375**	,455**	,394**	1,000	,769**	,449**	,109
		Sig. (2-tailed)	,203	,080	,302	,905	,020	,313	,019	,000	,000	,000	.	,000	,000	,281
		N	100	100	100	100	100	100	100	100	100	100	100	100	100	100
	GS5	Correlation Coefficient	,126	,150	,148	,182	,188	,179	,243*	,366**	,623**	,490**	,769**	1,000	,471**	,115
		Sig. (2-tailed)	,212	,136	,142	,069	,061	,074	,015	,000	,000	,000	,000	.	,000	,254
		N	100	100	100	100	100	100	100	100	100	100	100	100	100	100
	GS6	Correlation Coefficient	,145	-,013	,216*	,112	,253*	,181	,257**	,218*	,480**	,368**	,449**	,471**	1,000	,368**
		Sig. (2-tailed)	,150	,896	,031	,268	,011	,072	,010	,029	,000	,000	,000	,000	.	,000
		N	100	100	100	100	100	100	100	100	100	100	100	100	100	100
	GS7	Correlation Coefficient	,154	,153	,309**	,280**	,327**	,278**	,248*	,081	,086	,083	,109	,115	,368**	1,000
		Sig. (2-tailed)	,126	,128	,002	,005	,001	,005	,013	,424	,397	,413	,281	,254	,000	.
		N	100	100	100	100	100	100	100	100	100	100	100	100	100	100
	GE1	Correlation Coefficient	,381**	,339**	,267**	,550**	,307**	,459**	,345**	,027	,093	,095	,092	,066	,146	,354**
		Sig. (2-tailed)	,000	,001	,007	,000	,002	,000	,000	,791	,356	,348	,360	,515	,147	,000
		N	100	100	100	100	100	100	100	100	100	100	100	100	100	100
	GE2	Correlation Coefficient	,118	,126	,121	,106	,144	,204*	,323**	,428**	,371**	,343**	,353**	,248*	,184	,254*
		Sig. (2-tailed)	,241	,212	,230	,294	,154	,042	,001	,000	,000	,000	,000	,013	,067	,011
		N	100	100	100	100	100	100	100	100	100	100	100	100	100	100
	GE3	Correlation Coefficient	,147	,097	,140	,233*	,265**	,233*	,148	,084	,010	,074	,046	,046	-,019	,055
		Sig. (2-tailed)	,144	,339	,165	,019	,008	,019	,142	,407	,922	,462	,647	,651	,852	,590
		N	100	100	100	100	100	100	100	100	100	100	100	100	100	100
	GE4	Correlation Coefficient	,267**	,200*	,185	,500**	,197*	,206*	,189	,076	,197*	,104	,068	,071	,129	,310**
		Sig. (2-tailed)	,007	,046	,065	,000	,049	,040	,059	,449	,049	,305	,502	,483	,202	,002
		N	100	100	100	100	100	100	100	100	100	100	100	100	100	100
	GE5	Correlation Coefficient	,270**	,217*	,108	,397**	,190	,265**	,208*	,149	,104	,261**	,016	,026	-,055	-,007
		Sig. (2-tailed)	,007	,030	,285	,000	,058	,008	,038	,138	,303	,009	,872	,795	,588	,942
		N	100	100	100	100	100	100	100	100	100	100	100	100	100	100
	GE6	Correlation Coefficient	,445**	,299**	,275**	,416**	,318**	,460**	,365**	,031	,151	,113	,193	,215*	,120	,229*
		Sig. (2-tailed)	,000	,003	,006	,000	,001	,000	,000	,756	,132	,262	,055	,032	,234	,022
		N	100	100	100	100	100	100	100	100	100	100	100	100	100	100
	GF1	Correlation Coefficient	,489**	,337**	,524**	,450**	,241*	,415**	,465**	,416**	,279**	,375**	,232*	,335**	,360**	,330**
		Sig. (2-tailed)	,000	,001	,000	,000	,016	,000	,000	,000	,005	,000	,020	,001	,000	,001
		N	100	100	100	100	100	100	100	100	100	100	100	100	100	100
	GF2	Correlation Coefficient	,431**	,254*	,417**	,376**	,218*	,396**	,341**	,392**	,304**	,410**	,245*	,388**	,344**	,267**
		Sig. (2-tailed)	,000	,011	,000	,000	,030	,000	,001	,000	,002	,000	,014	,000	,000	,007
		N	100	100	100	100	100	100	100	100	100	100	100	100	100	100
	GF3	Correlation Coefficient	,313**	,143	,219*	,204*	,163	,276**	,355**	,495**	,442**	,470**	,365**	,374**	,249*	,243*
		Sig. (2-tailed)	,002	,157	,029	,042	,105	,005	,000	,000	,000	,000	,000	,000	,013	,015
		N	100	100	100	100	100	100	100	100	100	100	100	100	100	100
	GF4	Correlation Coefficient	,123	,195	,117	,114	,130	,253*	,164	,437**	,372**	,395**	,613**	,583**	,217*	,133
		Sig. (2-tailed)	,223	,052	,247	,260	,196	,011	,104	,000	,000	,000	,000	,000	,030	,187
		N	100	100	100	100	100	100	100	100	100	100	100	100	100	100
	GF5	Correlation Coefficient	,280**	,120	,169	,258**	,168	,191	,154	,139	,280**	,200*	,295**	,397**	,234*	,118
		Sig. (2-tailed)	,005	,235	,094	,010	,095	,057	,127	,166	,005	,046	,003	,000	,019	,243
		N	100	100	100	100	100	100	100	100	100	100	100	100	100	100
	GF6	Correlation Coefficient	,292**	,072	,147	,156	,144	,290**	,236*	,437**	,375**	,456**	,293**	,410**	,295**	,146
		Sig. (2-tailed)	,003	,479	,145	,120	,153	,003	,018	,000	,000	,000	,003	,000	,003	,146
		N	100	100	100	100	100	100	100	100	100	100	100	100	100	100
	GF7	Correlation Coefficient	,366**	,075	,281**	,351**	,198*	,459**	,290**	,351**	,336**	,331**	,313**	,431**	,237*	,303**
		Sig. (2-tailed)	,000	,458	,005	,000	,048	,000	,003	,000	,001	,001	,001	,000	,017	,002
		N	100	100	100	100	100	100	100	100	100	100	100	100	100	100
	CX1	Correlation Coefficient	,140	,099	,288**	,299**	,023	,231*	,040	,027	-,054	,057	-,057	,037	-,024	,173
		Sig. (2-tailed)	,164	,326	,004	,003	,818	,021	,689	,791	,590	,571	,574	,715	,813	,086
		N	100	100	100	100	100	100	100	100	100	100	100	100	100	100
	CX2	Correlation Coefficient	,161	,130	,195	,298**	,114	-,001	,032	,035	,007	,043	-,153	-,048	-,173	,156
		Sig. (2-tailed)	,109	,198	,052	,003	,261	,995	,752	,730	,942	,671	,130	,637	,085	,121
		N	100	100	100	100	100	100	100	100	100	100	100	100	100	100
	CX3	Correlation Coefficient	,132	,026	,213*	,223*	,359**	,093	,005	,107	,153	,167	,101	,115	,142	,354**
		Sig. (2-tailed)	,192	,798	,033	,026	,000	,360	,961	,289	,128	,096	,319	,255	,159	,000
		N	100	100	100	100	100	100	100	100	100	100	100	100	100	100
	CX4	Correlation Coefficient	,386**	,213*	,230*	,353**	,434**	,251*	,146	,090	,174	,119	,154	,187	,183	,296**
		Sig. (2-tailed)	,000	,034	,021	,000	,000	,012	,149	,376	,084	,240	,127	,063	,068	,003
		N	100	100	100	100	100	100	100	100	100	100	100	100	100	100
	CX5	Correlation Coefficient	,111	,071	,167	,213*	,343**	,339**	,206*	,058	-,084	-,087	,180	,104	,037	,159
		Sig. (2-tailed)	,270	,485	,097	,033	,000	,001	,040	,567	,408	,387	,074	,304	,713	,113
		N	100	100	100	100	100	100	100	100	100	100	100	100	100	100
	CX6	Correlation Coefficient	,088	,063	,213*	,253*	,095	,183	,151	-,106	-,056	-,041	,054	,023	,184	,121
		Sig. (2-tailed)	,385	,532	,033	,011	,348	,068	,134	,295	,583	,686	,594	,818	,067	,229
		N	100	100	100	100	100	100	100	100	100	100	100	100	100	100
	CX7	Correlation Coefficient	,174	,360**	,404**	,231*	,278**	,200*	,147	,039	-,025	-,057	-,011	,085	,071	,250*
		Sig. (2-tailed)	,084	,000	,000	,021	,005	,047	,143	,697	,807	,573	,912	,398	,482	,012
		N	100	100	100	100	100	100	100	100	100	100	100	100	100	100
	B4	Correlation Coefficient	,161	,114	,290**	,216*	,194	,274**	,161	,245*	,221*	,265**	,025	,189	,094	,364**
		Sig. (2-tailed)	,109	,258	,003	,031	,052	,006	,110	,014	,027	,008	,806	,060	,351	,000
		N	100	100	100	100	100	100	100	100	100	100	100	100	100	100
	BMT7	Correlation Coefficient	,128	,040	,021	,081	,026	,104	,198*	-,092	-,045	-,068	,061	,039	-,087	,009
		Sig. (2-tailed)	,206	,691	,837	,425	,796	,301	,048	,361	,658	,501	,548	,699	,387	,926
		N	100	100	100	100	100	100	100	100	100	100	100	100	100	100
	BL4	Correlation Coefficient	,187	,179	,198*	,213*	,177	,103	,142	,261**	,258**	,400**	,147	,143	,209*	,416**
		Sig. (2-tailed)	,063	,075	,048	,033	,079	,308	,158	,009	,009	,000	,145	,156	,037	,000
		N	100	100	100	100	100	100	100	100	100	100	100	100	100	100
	C7	Correlation Coefficient	,136	,152	,160	,214*	,181	,150	,140	,224*	,164	,316**	,114	,180	,141	,229*
		Sig. (2-tailed)	,178	,131	,111	,033	,071	,136	,166	,025	,103	,001	,257	,074	,162	,022
		N	100	100	100	100	100	100	100	100	100	100	100	100	100	100
	C6	Correlation Coefficient	,287**	,259**	,270**	,277**	-,044	,312**	,182	,166	,129	,017	,239*	,230*	,048	,161
		Sig. (2-tailed)	,004	,009	,007	,005	,663	,002	,069	,099	,201	,866	,017	,021	,632	,109
		N	100	100	100	100	100	100	100	100	100	100	100	100	100	100
	BL1	Correlation Coefficient	,171	,279**	,158	,319**	,229*	,001	,067	,000	,086	,137	-,044	-,032	,038	,254*
		Sig. (2-tailed)	,089	,005	,117	,001	,022	,993	,507	,998	,396	,174	,667	,755	,707	,011
		N	100	100	100	100	100	100	100	100	100	100	100	100	100	100
	BL3	Correlation Coefficient	-,011	,165	,298**	,273**	,380**	,191	,109	,029	,023	,020	-,092	,092	,091	,212*
		Sig. (2-tailed)	,912	,102	,003	,006	,000	,057	,279	,772	,817	,842	,364	,361	,366	,034
		N	100	100	100	100	100	100	100	100	100	100	100	100	100	100
	HN1	Correlation Coefficient	,162	,143	,282**	,136	,162	,184	,278**	,142	,162	,162	,175	,273**	,079	,150
		Sig. (2-tailed)	,106	,156	,005	,177	,108	,067	,005	,158	,106	,108	,081	,006	,436	,137
		N	100	100	100	100	100	100	100	100	100	100	100	100	100	100

			GE1	GE2	GE3	GE4	GE5	GE6	GF1	GF2	GF3	GF4	GF5	GF6	GF7	CX1
Spearman's rho	GP1	Correlation Coefficient	,381**	,118	,147	,267**	,270**	,445**	,489**	,431**	,313**	,123	,280**	,292**	,366**	,140
		Sig. (2-tailed)	,000	,241	,144	,007	,007	,000	,000	,000	,002	,223	,005	,003	,000	,164
		N	100	100	100	100	100	100	100	100	100	100	100	100	100	100
	GP2	Correlation Coefficient	,339**	,126	,097	,200*	,217*	,299**	,337**	,254*	,143	,195	,120	,072	,075	,099
		Sig. (2-tailed)	,001	,212	,339	,046	,030	,003	,001	,011	,157	,052	,235	,479	,458	,326
		N	100	100	100	100	100	100	100	100	100	100	100	100	100	100
	GP3	Correlation Coefficient	,267**	,121	,140	,185	,108	,275**	,524**	,417**	,219*	,117	,169	,147	,281**	,288**
		Sig. (2-tailed)	,007	,230	,165	,065	,285	,006	,000	,000	,029	,247	,094	,145	,005	,004
		N	100	100	100	100	100	100	100	100	100	100	100	100	100	100
	GP4	Correlation Coefficient	,550**	,106	,233*	,500**	,397**	,416**	,450**	,376**	,204*	,114	,258**	,156	,351**	,299**
		Sig. (2-tailed)	,000	,294	,019	,000	,000	,000	,000	,000	,042	,260	,010	,120	,000	,003
		N	100	100	100	100	100	100	100	100	100	100	100	100	100	100
	GP5	Correlation Coefficient	,307**	,144	,265**	,197*	,190	,318**	,241*	,218*	,163	,130	,168	,144	,198*	,023
		Sig. (2-tailed)	,002	,154	,008	,049	,058	,001	,016	,030	,105	,196	,095	,153	,048	,818
		N	100	100	100	100	100	100	100	100	100	100	100	100	100	100
	GP6	Correlation Coefficient	,459**	,204*	,233*	,206*	,265**	,460**	,415**	,396**	,276**	,253*	,191	,290**	,459**	,231*
		Sig. (2-tailed)	,000	,042	,019	,040	,008	,000	,000	,000	,005	,011	,057	,003	,000	,021
		N	100	100	100	100	100	100	100	100	100	100	100	100	100	100
	GP7	Correlation Coefficient	,345**	,323**	,148	,189	,208*	,365**	,465**	,341**	,355**	,164	,154	,236*	,290**	,040
		Sig. (2-tailed)	,000	,001	,142	,059	,038	,000	,000	,001	,000	,104	,127	,018	,003	,689
		N	100	100	100	100	100	100	100	100	100	100	100	100	100	100
	GS1	Correlation Coefficient	,027	,428**	,084	,076	,149	,031	,416**	,392**	,495**	,437**	,139	,437**	,351**	,027
		Sig. (2-tailed)	,791	,000	,407	,449	,138	,756	,000	,000	,000	,000	,166	,000	,000	,791
		N	100	100	100	100	100	100	100	100	100	100	100	100	100	100
	GS2	Correlation Coefficient	,093	,371**	,010	,197*	,104	,151	,279**	,304**	,442**	,372**	,280**	,375**	,336**	-,054
		Sig. (2-tailed)	,356	,000	,922	,049	,303	,132	,005	,002	,000	,000	,005	,000	,001	,590
		N	100	100	100	100	100	100	100	100	100	100	100	100	100	100
	GS3	Correlation Coefficient	,095	,343**	,074	,104	,261**	,113	,375**	,410**	,470**	,395**	,200*	,456**	,331**	,057
		Sig. (2-tailed)	,348	,000	,462	,305	,009	,262	,000	,000	,000	,000	,046	,000	,001	,571
		N	100	100	100	100	100	100	100	100	100	100	100	100	100	100
	GS4	Correlation Coefficient	,092	,353**	,046	,068	,016	,193	,232*	,245*	,365**	,613**	,295**	,293**	,313**	-,057
		Sig. (2-tailed)	,360	,000	,647	,502	,872	,055	,020	,014	,000	,000	,003	,003	,001	,574
		N	100	100	100	100	100	100	100	100	100	100	100	100	100	100
	GS5	Correlation Coefficient	,066	,248*	,046	,071	,026	,215*	,335**	,388**	,374**	,583**	,397**	,410**	,431**	,037
		Sig. (2-tailed)	,515	,013	,651	,483	,795	,032	,001	,000	,000	,000	,000	,000	,000	,715
		N	100	100	100	100	100	100	100	100	100	100	100	100	100	100
	GS6	Correlation Coefficient	,146	,184	-,019	,129	-,055	,120	,360**	,344**	,249*	,217*	,234*	,295**	,237*	-,024
		Sig. (2-tailed)	,147	,067	,852	,202	,588	,234	,000	,000	,013	,030	,019	,003	,017	,813
		N	100	100	100	100	100	100	100	100	100	100	100	100	100	100
	GS7	Correlation Coefficient	,354**	,254*	,055	,310**	-,007	,229*	,330**	,267**	,243*	,133	,118	,146	,303**	,173
		Sig. (2-tailed)	,000	,011	,590	,002	,942	,022	,001	,007	,015	,187	,243	,146	,002	,086
		N	100	100	100	100	100	100	100	100	100	100	100	100	100	100
	GE1	Correlation Coefficient	1,000	,196	,137	,572**	,203*	,339**	,306**	,260**	,216*	,117	,297**	,238*	,359**	,203*
		Sig. (2-tailed)	.	,051	,174	,000	,043	,001	,002	,009	,031	,247	,003	,017	,000	,042
		N	100	100	100	100	100	100	100	100	100	100	100	100	100	100
	GE2	Correlation Coefficient	,196	1,000	,020	,101	,110	,231*	,353**	,304**	,476**	,529**	,205*	,377**	,263**	,006
		Sig. (2-tailed)	,051	.	,844	,317	,275	,021	,000	,002	,000	,000	,041	,000	,008	,952
		N	100	100	100	100	100	100	100	100	100	100	100	100	100	100
	GE3	Correlation Coefficient	,137	,020	1,000	,114	,180	,241*	,214*	,175	,153	,079	,016	,023	,145	,025
		Sig. (2-tailed)	,174	,844	.	,257	,073	,016	,033	,081	,129	,435	,875	,817	,150	,802
		N	100	100	100	100	100	100	100	100	100	100	100	100	100	100
	GE4	Correlation Coefficient	,572**	,101	,114	1,000	,196	,190	,150	,117	,126	-,007	,183	,063	,275**	,209*
		Sig. (2-tailed)	,000	,317	,257	.	,051	,058	,137	,247	,211	,947	,069	,530	,006	,037
		N	100	100	100	100	100	100	100	100	100	100	100	100	100	100
	GE5	Correlation Coefficient	,203*	,110	,180	,196	1,000	,507**	,131	,152	,149	,138	,078	,096	,261**	,298**
		Sig. (2-tailed)	,043	,275	,073	,051	.	,000	,194	,132	,140	,172	,443	,344	,009	,003
		N	100	100	100	100	100	100	100	100	100	100	100	100	100	100
	GE6	Correlation Coefficient	,339**	,231*	,241*	,190	,507**	1,000	,290**	,383**	,224*	,281**	,276**	,121	,393**	,431**
		Sig. (2-tailed)	,001	,021	,016	,058	,000	.	,003	,000	,025	,005	,005	,232	,000	,000
		N	100	100	100	100	100	100	100	100	100	100	100	100	100	100
	GF1	Correlation Coefficient	,306**	,353**	,214*	,150	,131	,290**	1,000	,778**	,590**	,389**	,230*	,444**	,374**	,091
		Sig. (2-tailed)	,002	,000	,033	,137	,194	,003	.	,000	,000	,000	,022	,000	,000	,366
		N	100	100	100	100	100	100	100	100	100	100	100	100	100	100
	GF2	Correlation Coefficient	,260**	,304**	,175	,117	,152	,383**	,778**	1,000	,631**	,373**	,212*	,380**	,396**	,246*
		Sig. (2-tailed)	,009	,002	,081	,247	,132	,000	,000	.	,000	,000	,035	,000	,000	,014
		N	100	100	100	100	100	100	100	100	100	100	100	100	100	100
	GF3	Correlation Coefficient	,216*	,476**	,153	,126	,149	,224*	,590**	,631**	1,000	,530**	,204*	,499**	,420**	-,050
		Sig. (2-tailed)	,031	,000	,129	,211	,140	,025	,000	,000	.	,000	,041	,000	,000	,622
		N	100	100	100	100	100	100	100	100	100	100	100	100	100	100
	GF4	Correlation Coefficient	,117	,529**	,079	-,007	,138	,281**	,389**	,373**	,530**	1,000	,147	,422**	,347**	,129
		Sig. (2-tailed)	,247	,000	,435	,947	,172	,005	,000	,000	,000	.	,146	,000	,000	,202
		N	100	100	100	100	100	100	100	100	100	100	100	100	100	100
	GF5	Correlation Coefficient	,297**	,205*	,016	,183	,078	,276**	,230*	,212*	,204*	,147	1,000	,405**	,521**	,249*
		Sig. (2-tailed)	,003	,041	,875	,069	,443	,005	,022	,035	,041	,146	.	,000	,000	,012
		N	100	100	100	100	100	100	100	100	100	100	100	100	100	100
	GF6	Correlation Coefficient	,238*	,377**	,023	,063	,096	,121	,444**	,380**	,499**	,422**	,405**	1,000	,563**	,071
		Sig. (2-tailed)	,017	,000	,817	,530	,344	,232	,000	,000	,000	,000	,000	.	,000	,486
		N	100	100	100	100	100	100	100	100	100	100	100	100	100	100
	GF7	Correlation Coefficient	,359**	,263**	,145	,275**	,261**	,393**	,374**	,396**	,420**	,347**	,521**	,563**	1,000	,259**
		Sig. (2-tailed)	,000	,008	,150	,006	,009	,000	,000	,000	,000	,000	,000	,000	.	,009
		N	100	100	100	100	100	100	100	100	100	100	100	100	100	100
	CX1	Correlation Coefficient	,203*	,006	,025	,209*	,298**	,431**	,091	,246*	-,050	,129	,249*	,071	,259**	1,000
		Sig. (2-tailed)	,042	,952	,802	,037	,003	,000	,366	,014	,622	,202	,012	,486	,009	.
		N	100	100	100	100	100	100	100	100	100	100	100	100	100	100
	CX2	Correlation Coefficient	,263**	-,015	-,009	,241*	,220*	,183	,041	,107	-,014	-,066	,179	,056	,122	,531**
		Sig. (2-tailed)	,008	,883	,932	,016	,028	,068	,685	,288	,889	,514	,075	,581	,228	,000
		N	100	100	100	100	100	100	100	100	100	100	100	100	100	100
	CX3	Correlation Coefficient	,074	,094	,010	,247*	,191	,097	,197*	,157	,123	,040	,057	,071	,156	,256*
		Sig. (2-tailed)	,466	,350	,923	,013	,057	,335	,050	,119	,224	,691	,576	,485	,122	,010
		N	100	100	100	100	100	100	100	100	100	100	100	100	100	100
	CX4	Correlation Coefficient	,374**	,171	,059	,228*	,180	,277**	,162	,250*	,157	,233*	,181	,144	,271**	,203*
		Sig. (2-tailed)	,000	,089	,558	,023	,074	,005	,107	,012	,118	,020	,072	,152	,006	,043
		N	100	100	100	100	100	100	100	100	100	100	100	100	100	100
	CX5	Correlation Coefficient	,142	,161	,036	,042	,147	,384**	,112	,226*	,092	,218*	,042	,012	,128	,341**
		Sig. (2-tailed)	,159	,109	,720	,680	,145	,000	,267	,024	,361	,029	,682	,902	,205	,001
		N	100	100	100	100	100	100	100	100	100	100	100	100	100	100
	CX6	Correlation Coefficient	,300**	-,075	,056	,201*	-,016	,238*	,139	,152	-,092	-,090	,138	-,063	,087	,406**
		Sig. (2-tailed)	,002	,457	,583	,045	,878	,017	,167	,131	,365	,373	,172	,532	,388	,000
		N	100	100	100	100	100	100	100	100	100	100	100	100	100	100
	CX7	Correlation Coefficient	,184	,100	,045	,169	,046	,257**	,242*	,136	,118	,161	,159	,079	,035	,253*
		Sig. (2-tailed)	,066	,320	,655	,093	,646	,010	,015	,177	,241	,109	,113	,437	,733	,011
		N	100	100	100	100	100	100	100	100	100	100	100	100	100	100
	B4	Correlation Coefficient	,100	,270**	,121	,006	,054	,217*	,388**	,301**	,355**	,176	,026	,175	,281**	,034
		Sig. (2-tailed)	,322	,007	,231	,950	,590	,030	,000	,002	,000	,080	,800	,081	,005	,739
		N	100	100	100	100	100	100	100	100	100	100	100	100	100	100
	BMT7	Correlation Coefficient	,086	,184	-,008	-,043	,157	,362**	-,011	,196	,143	,157	,107	,044	,102	,211*
		Sig. (2-tailed)	,397	,067	,933	,674	,118	,000	,916	,051	,157	,119	,289	,665	,312	,035
		N	100	100	100	100	100	100	100	100	100	100	100	100	100	100
	BL4	Correlation Coefficient	,069	,177	-,021	,052	,073	,067	,315**	,361**	,417**	,178	,107	,192	,197	-,044
		Sig. (2-tailed)	,495	,078	,839	,606	,472	,507	,001	,000	,000	,076	,287	,056	,050	,661
		N	100	100	100	100	100	100	100	100	100	100	100	100	100	100
	C7	Correlation Coefficient	,174	,223*	-,004	,104	,161	,141	,227*	,231*	,321**	,211*	,219*	,239*	,259**	,120
		Sig. (2-tailed)	,084	,026	,965	,303	,109	,161	,023	,021	,001	,035	,028	,017	,009	,234
		N	100	100	100	100	100	100	100	100	100	100	100	100	100	100
	C6	Correlation Coefficient	,216*	,155	,061	,051	,039	,152	,364**	,308**	,278**	,302**	,319**	,170	,321**	,154
		Sig. (2-tailed)	,031	,123	,544	,613	,698	,132	,000	,002	,005	,002	,001	,090	,001	,127
		N	100	100	100	100	100	100	100	100	100	100	100	100	100	100
	BL1	Correlation Coefficient	,261**	,044	-,028	,315**	,139	,105	,080	-,026	,054	,058	,034	-,126	-,052	,139
		Sig. (2-tailed)	,009	,665	,782	,001	,168	,298	,428	,800	,592	,568	,738	,212	,605	,166
		N	100	100	100	100	100	100	100	100	100	100	100	100	100	100
	BL3	Correlation Coefficient	,186	,127	,115	,192	,157	,140	,170	,187	,110	,081	,160	,066	,101	,251*
		Sig. (2-tailed)	,065	,208	,255	,055	,120	,164	,091	,062	,276	,425	,111	,512	,316	,012
		N	100	100	100	100	100	100	100	100	100	100	100	100	100	100
	HN1	Correlation Coefficient	,122	,267**	-,060	-,030	-,020	,139	,391**	,405**	,404**	,370**	,042	,235*	,250*	,006
		Sig. (2-tailed)	,225	,007	,552	,768	,845	,169	,000	,000	,000	,000	,675	,019	,012	,952
		N	100	100	100	100	100	100	100	100	100	100	100	100	100	100

			CX2	CX3	CX4	CX5	CX6	CX7	B4	BMT7	BL4	C7	C6	BL1	BL3	HN1
Spearman's rho	GP1	Correlation Coefficient	,161	,132	,386**	,111	,088	,174	,161	,128	,187	,136	,287**	,171	-,011	,162
		Sig. (2-tailed)	,109	,192	,000	,270	,385	,084	,109	,206	,063	,178	,004	,089	,912	,106
		N	100	100	100	100	100	100	100	100	100	100	100	100	100	100
	GP2	Correlation Coefficient	,130	,026	,213*	,071	,063	,360**	,114	,040	,179	,152	,259**	,279**	,165	,143
		Sig. (2-tailed)	,198	,798	,034	,485	,532	,000	,258	,691	,075	,131	,009	,005	,102	,156
		N	100	100	100	100	100	100	100	100	100	100	100	100	100	100
	GP3	Correlation Coefficient	,195	,213*	,230*	,167	,213*	,404**	,290**	,021	,198*	,160	,270**	,158	,298**	,282**
		Sig. (2-tailed)	,052	,033	,021	,097	,033	,000	,003	,837	,048	,111	,007	,117	,003	,005
		N	100	100	100	100	100	100	100	100	100	100	100	100	100	100
	GP4	Correlation Coefficient	,298**	,223*	,353**	,213*	,253*	,231*	,216*	,081	,213*	,214*	,277**	,319**	,273**	,136
		Sig. (2-tailed)	,003	,026	,000	,033	,011	,021	,031	,425	,033	,033	,005	,001	,006	,177
		N	100	100	100	100	100	100	100	100	100	100	100	100	100	100
	GP5	Correlation Coefficient	,114	,359**	,434**	,343**	,095	,278**	,194	,026	,177	,181	-,044	,229*	,380**	,162
		Sig. (2-tailed)	,261	,000	,000	,000	,348	,005	,052	,796	,079	,071	,663	,022	,000	,108
		N	100	100	100	100	100	100	100	100	100	100	100	100	100	100
	GP6	Correlation Coefficient	-,001	,093	,251*	,339**	,183	,200*	,274**	,104	,103	,150	,312**	,001	,191	,184
		Sig. (2-tailed)	,995	,360	,012	,001	,068	,047	,006	,301	,308	,136	,002	,993	,057	,067
		N	100	100	100	100	100	100	100	100	100	100	100	100	100	100
	GP7	Correlation Coefficient	,032	,005	,146	,206*	,151	,147	,161	,198*	,142	,140	,182	,067	,109	,278**
		Sig. (2-tailed)	,752	,961	,149	,040	,134	,143	,110	,048	,158	,166	,069	,507	,279	,005
		N	100	100	100	100	100	100	100	100	100	100	100	100	100	100
	GS1	Correlation Coefficient	,035	,107	,090	,058	-,106	,039	,245*	-,092	,261**	,224*	,166	,000	,029	,142
		Sig. (2-tailed)	,730	,289	,376	,567	,295	,697	,014	,361	,009	,025	,099	,998	,772	,158
		N	100	100	100	100	100	100	100	100	100	100	100	100	100	100
	GS2	Correlation Coefficient	,007	,153	,174	-,084	-,056	-,025	,221*	-,045	,258**	,164	,129	,086	,023	,162
		Sig. (2-tailed)	,942	,128	,084	,408	,583	,807	,027	,658	,009	,103	,201	,396	,817	,106
		N	100	100	100	100	100	100	100	100	100	100	100	100	100	100
	GS3	Correlation Coefficient	,043	,167	,119	-,087	-,041	-,057	,265**	-,068	,400**	,316**	,017	,137	,020	,162
		Sig. (2-tailed)	,671	,096	,240	,387	,686	,573	,008	,501	,000	,001	,866	,174	,842	,108
		N	100	100	100	100	100	100	100	100	100	100	100	100	100	100
	GS4	Correlation Coefficient	-,153	,101	,154	,180	,054	-,011	,025	,061	,147	,114	,239*	-,044	-,092	,175
		Sig. (2-tailed)	,130	,319	,127	,074	,594	,912	,806	,548	,145	,257	,017	,667	,364	,081
		N	100	100	100	100	100	100	100	100	100	100	100	100	100	100
	GS5	Correlation Coefficient	-,048	,115	,187	,104	,023	,085	,189	,039	,143	,180	,230*	-,032	,092	,273**
		Sig. (2-tailed)	,637	,255	,063	,304	,818	,398	,060	,699	,156	,074	,021	,755	,361	,006
		N	100	100	100	100	100	100	100	100	100	100	100	100	100	100
	GS6	Correlation Coefficient	-,173	,142	,183	,037	,184	,071	,094	-,087	,209*	,141	,048	,038	,091	,079
		Sig. (2-tailed)	,085	,159	,068	,713	,067	,482	,351	,387	,037	,162	,632	,707	,366	,436
		N	100	100	100	100	100	100	100	100	100	100	100	100	100	100
	GS7	Correlation Coefficient	,156	,354**	,296**	,159	,121	,250*	,364**	,009	,416**	,229*	,161	,254*	,212*	,150
		Sig. (2-tailed)	,121	,000	,003	,113	,229	,012	,000	,926	,000	,022	,109	,011	,034	,137
		N	100	100	100	100	100	100	100	100	100	100	100	100	100	100
	GE1	Correlation Coefficient	,263**	,074	,374**	,142	,300**	,184	,100	,086	,069	,174	,216*	,261**	,186	,122
		Sig. (2-tailed)	,008	,466	,000	,159	,002	,066	,322	,397	,495	,084	,031	,009	,065	,225
		N	100	100	100	100	100	100	100	100	100	100	100	100	100	100
	GE2	Correlation Coefficient	-,015	,094	,171	,161	-,075	,100	,270**	,184	,177	,223*	,155	,044	,127	,267**
		Sig. (2-tailed)	,883	,350	,089	,109	,457	,320	,007	,067	,078	,026	,123	,665	,208	,007
		N	100	100	100	100	100	100	100	100	100	100	100	100	100	100
	GE3	Correlation Coefficient	-,009	,010	,059	,036	,056	,045	,121	-,008	-,021	-,004	,061	-,028	,115	-,060
		Sig. (2-tailed)	,932	,923	,558	,720	,583	,655	,231	,933	,839	,965	,544	,782	,255	,552
		N	100	100	100	100	100	100	100	100	100	100	100	100	100	100
	GE4	Correlation Coefficient	,241*	,247*	,228*	,042	,201*	,169	,006	-,043	,052	,104	,051	,315**	,192	-,030
		Sig. (2-tailed)	,016	,013	,023	,680	,045	,093	,950	,674	,606	,303	,613	,001	,055	,768
		N	100	100	100	100	100	100	100	100	100	100	100	100	100	100
	GE5	Correlation Coefficient	,220*	,191	,180	,147	-,016	,046	,054	,157	,073	,161	,039	,139	,157	-,020
		Sig. (2-tailed)	,028	,057	,074	,145	,878	,646	,590	,118	,472	,109	,698	,168	,120	,845
		N	100	100	100	100	100	100	100	100	100	100	100	100	100	100
	GE6	Correlation Coefficient	,183	,097	,277**	,384**	,238*	,257**	,217*	,362**	,067	,141	,152	,105	,140	,139
		Sig. (2-tailed)	,068	,335	,005	,000	,017	,010	,030	,000	,507	,161	,132	,298	,164	,169
		N	100	100	100	100	100	100	100	100	100	100	100	100	100	100
	GF1	Correlation Coefficient	,041	,197*	,162	,112	,139	,242*	,388**	-,011	,315**	,227*	,364**	,080	,170	,391**
		Sig. (2-tailed)	,685	,050	,107	,267	,167	,015	,000	,916	,001	,023	,000	,428	,091	,000
		N	100	100	100	100	100	100	100	100	100	100	100	100	100	100
	GF2	Correlation Coefficient	,107	,157	,250*	,226*	,152	,136	,301**	,196	,361**	,231*	,308**	-,026	,187	,405**
		Sig. (2-tailed)	,288	,119	,012	,024	,131	,177	,002	,051	,000	,021	,002	,800	,062	,000
		N	100	100	100	100	100	100	100	100	100	100	100	100	100	100
	GF3	Correlation Coefficient	-,014	,123	,157	,092	-,092	,118	,355**	,143	,417**	,321**	,278**	,054	,110	,404**
		Sig. (2-tailed)	,889	,224	,118	,361	,365	,241	,000	,157	,000	,001	,005	,592	,276	,000
		N	100	100	100	100	100	100	100	100	100	100	100	100	100	100
	GF4	Correlation Coefficient	-,066	,040	,233*	,218*	-,090	,161	,176	,157	,178	,211*	,302**	,058	,081	,370**
		Sig. (2-tailed)	,514	,691	,020	,029	,373	,109	,080	,119	,076	,035	,002	,568	,425	,000
		N	100	100	100	100	100	100	100	100	100	100	100	100	100	100
	GF5	Correlation Coefficient	,179	,057	,181	,042	,138	,159	,026	,107	,107	,219*	,319**	,034	,160	,042
		Sig. (2-tailed)	,075	,576	,072	,682	,172	,113	,800	,289	,287	,028	,001	,738	,111	,675
		N	100	100	100	100	100	100	100	100	100	100	100	100	100	100
	GF6	Correlation Coefficient	,056	,071	,144	,012	-,063	,079	,175	,044	,192	,239*	,170	-,126	,066	,235*
		Sig. (2-tailed)	,581	,485	,152	,902	,532	,437	,081	,665	,056	,017	,090	,212	,512	,019
		N	100	100	100	100	100	100	100	100	100	100	100	100	100	100
	GF7	Correlation Coefficient	,122	,156	,271**	,128	,087	,035	,281**	,102	,197	,259**	,321**	-,052	,101	,250*
		Sig. (2-tailed)	,228	,122	,006	,205	,388	,733	,005	,312	,050	,009	,001	,605	,316	,012
		N	100	100	100	100	100	100	100	100	100	100	100	100	100	100
	CX1	Correlation Coefficient	,531**	,256*	,203*	,341**	,406**	,253*	,034	,211*	-,044	,120	,154	,139	,251*	,006
		Sig. (2-tailed)	,000	,010	,043	,001	,000	,011	,739	,035	,661	,234	,127	,166	,012	,952
		N	100	100	100	100	100	100	100	100	100	100	100	100	100	100
	CX2	Correlation Coefficient	1,000	,240*	,311**	,088	,303**	,357**	-,005	,313**	-,010	,144	-,032	,295**	,369**	-,005
		Sig. (2-tailed)	.	,016	,002	,383	,002	,000	,961	,002	,919	,154	,754	,003	,000	,957
		N	100	100	100	100	100	100	100	100	100	100	100	100	100	100
	CX3	Correlation Coefficient	,240*	1,000	,208*	,160	,185	,119	,160	,026	,212*	,215*	,009	,128	,263**	,064
		Sig. (2-tailed)	,016	.	,038	,113	,065	,239	,111	,794	,034	,032	,929	,203	,008	,527
		N	100	100	100	100	100	100	100	100	100	100	100	100	100	100
	CX4	Correlation Coefficient	,311**	,208*	1,000	,344**	,060	,285**	,140	,095	,043	,115	-,012	,196	,316**	,028
		Sig. (2-tailed)	,002	,038	.	,000	,551	,004	,165	,347	,670	,253	,905	,051	,001	,781
		N	100	100	100	100	100	100	100	100	100	100	100	100	100	100
	CX5	Correlation Coefficient	,088	,160	,344**	1,000	,216*	,295**	,102	,171	-,065	,026	,150	,039	,230*	,058
		Sig. (2-tailed)	,383	,113	,000	.	,031	,003	,315	,090	,521	,800	,137	,700	,022	,568
		N	100	100	100	100	100	100	100	100	100	100	100	100	100	100
	CX6	Correlation Coefficient	,303**	,185	,060	,216*	1,000	,208*	-,007	,194	-,092	,072	,180	,111	,181	,067
		Sig. (2-tailed)	,002	,065	,551	,031	.	,038	,945	,054	,363	,475	,074	,273	,072	,509
		N	100	100	100	100	100	100	100	100	100	100	100	100	100	100
	CX7	Correlation Coefficient	,357**	,119	,285**	,295**	,208*	1,000	,317**	,088	,057	,151	,156	,453**	,716**	,207*
		Sig. (2-tailed)	,000	,239	,004	,003	,038	.	,001	,385	,572	,134	,120	,000	,000	,039
		N	100	100	100	100	100	100	100	100	100	100	100	100	100	100
	B4	Correlation Coefficient	-,005	,160	,140	,102	-,007	,317**	1,000	-,134	,369**	,234*	,140	,085	,229*	,206*
		Sig. (2-tailed)	,961	,111	,165	,315	,945	,001	.	,182	,000	,019	,166	,401	,022	,039
		N	100	100	100	100	100	100	100	100	100	100	100	100	100	100
	BMT7	Correlation Coefficient	,313**	,026	,095	,171	,194	,088	-,134	1,000	-,044	,083	-,011	,102	,147	,199*
		Sig. (2-tailed)	,002	,794	,347	,090	,054	,385	,182	.	,665	,412	,913	,315	,143	,047
		N	100	100	100	100	100	100	100	100	100	100	100	100	100	100
	BL4	Correlation Coefficient	-,010	,212*	,043	-,065	-,092	,057	,369**	-,044	1,000	,318**	,279**	,156	,130	,245*
		Sig. (2-tailed)	,919	,034	,670	,521	,363	,572	,000	,665	.	,001	,005	,121	,198	,014
		N	100	100	100	100	100	100	100	100	100	100	100	100	100	100
	C7	Correlation Coefficient	,144	,215*	,115	,026	,072	,151	,234*	,083	,318**	1,000	,240*	,285**	,160	,366**
		Sig. (2-tailed)	,154	,032	,253	,800	,475	,134	,019	,412	,001	.	,016	,004	,113	,000
		N	100	100	100	100	100	100	100	100	100	100	100	100	100	100
	C6	Correlation Coefficient	-,032	,009	-,012	,150	,180	,156	,140	-,011	,279**	,240*	1,000	-,017	,011	,408**
		Sig. (2-tailed)	,754	,929	,905	,137	,074	,120	,166	,913	,005	,016	.	,867	,917	,000
		N	100	100	100	100	100	100	100	100	100	100	100	100	100	100
	BL1	Correlation Coefficient	,295**	,128	,196	,039	,111	,453**	,085	,102	,156	,285**	-,017	1,000	,385**	,110
		Sig. (2-tailed)	,003	,203	,051	,700	,273	,000	,401	,315	,121	,004	,867	.	,000	,277
		N	100	100	100	100	100	100	100	100	100	100	100	100	100	100
	BL3	Correlation Coefficient	,369**	,263**	,316**	,230*	,181	,716**	,229*	,147	,130	,160	,011	,385**	1,000	,203*
		Sig. (2-tailed)	,000	,008	,001	,022	,072	,000	,022	,143	,198	,113	,917	,000	.	,042
		N	100	100	100	100	100	100	100	100	100	100	100	100	100	100
	HN1	Correlation Coefficient	-,005	,064	,028	,058	,067	,207*	,206*	,199*	,245*	,366**	,408**	,110	,203*	1,000
		Sig. (2-tailed)	,957	,527	,781	,568	,509	,039	,039	,047	,014	,000	,000	,277	,042	.
		N	100	100	100	100	100	100	100	100	100	100	100	100	100	100

**. Correlation is significant at the 0.01 level (2-tailed).

*. Correlation is significant at the 0.05 level (2-tailed).
